# The prevalence of psychopathological symptoms in population during the COVID-19 pandemic

**DOI:** 10.1192/j.eurpsy.2021.748

**Published:** 2021-08-13

**Authors:** I. Belokrylov, T. Lineva, Y. Batyrev, A. Bocharnikova, A. Okuneva

**Affiliations:** Department Of Psychiatry And Medical Psychologi, Peoples Friendship University of Russia (RUDN University), Moscow, Russian Federation

## Abstract

**Introduction:**

The COVID-19 pandemic has caused significant lifestyle changes for the world’s population. The infection poses a threat to mental health due to direct invasion of the central nervous system of SARS-CoV-2, as well as as a source of mental stress associated, in particular, with the deformation of the structure of interpersonal communications under quarantine conditions.

**Objectives:**

The study was conducted to comparatively study the phenomenology and severity of psychopathological manifestations in quarantined and non-quarantined people during the COVID-19 pandemic.

**Methods:**

From April 18 to June 15, 2020, an online questionnaire “Symptom List 90” (SCL-90) was conducted among 837 adults in Russia, Kazakhstan, Belarus and other countries. 426 respondents were in strict home quarantine; 302 observed social distancing, but could go to work; 109 were not socially isolated.

**Results:**

There was a significant difference in the overall severity index (GSI) between strictly quarantine and non-quarantine groups with GSI values of 0.51 (0.24; 0.99) and 0.33 (0.16; 0.75), respectively (p = 0.001). Indicators of anxiety, depression, somatization, obsessive-compulsive symptoms, phobic anxiety, hostility and psychoticism were also significantly higher in quarantined individuals than in non-quarantined individuals (p <0.05).

**Conclusions:**

The results of the analysis indicate that in a pandemic, the most susceptible to psychopathological disorders are those living in the most severe quarantine, while the contingent whose lifestyle changes little under these conditions shows the best indicators of mental health. These data indicate the need to optimize the system of psychiatric preventive and curative care for the population in a pandemic.

**Conflict of interest:**

No significant relationships.

